# Institutional Notes and News

**Published:** 1910-12-10

**Authors:** 


### Institutional Notes and News.

On Monday last the monthly meeting of the Board of the Herefordshire General Hospital met to consider the report of a special committee, which had been appointed to consider certain suggestions and criticisms made by Sir Henry Burdett, who visited the institution earlier in the year. The special committee recommended the building of a new wing at a cost of some £8,000. If this is done it is felt that the county will have a memorial worthy alike of itself and of the late King. The new wing would form a complete hospital in itself, while the present building would be used for the administration. The discussion which followed revealed Lord Biddulph in favour and Mr. Albert Simpson against these proposals. It was decided finally that at the proposed public meeting, to be held for consideration of the form of the county memorial, Lord Biddulph and the Dean should support the hospital's claims. Thus ended an interesting debate, over which Sir Edward Hopton presided.

**Herefordshire General Hospital.**

A few weeks ago the Mayor of Wolverhampton convened a town's meeting for the purpose of considering a memorial to commemorate the reign of his late Majesty King Edward VII. The meeting decided unanimously that the memorial should take the form of a new wing at the Wolverhampton and Staffordshire General Hospital. The Board of Management of the General Hospital had already decided upon a scheme of reconstruction, and have the plans prepared. Included in this scheme is a new block of wards at the north-eastern end of the present building, and it is this block that is intended as the memorial. His Worship has issued an appeal for £6,000. The chairman of the Hospital Saturday Committee, recognising that no memorial could be complete unless the thousands occupied in the manufacturing and other industries of the town be given opportunities to contribute to the fund, convened a representative meeting of workmen, when, after discussion, it was decided as the most effective and economical means of raising money, to ask each employé throughout the town and district to contribute one penny per week for thirteen weeks. The matter is being taken up heartily, and the suggestions of the Committee are being carried out. It was felt that there would still be thousands of people who would not have the opportunity of contributing in the above manner. To meet these cases penny bricks have been issued in 5s. books. Hundreds of these books have been sent out, and, judging from the applications that are being constantly received, this system of raising money also will be both popular and lucrative.

**Wolverhampton General Hospital.**

Mr. William Sutton, Sen., in presenting his financial statement for the ten months ending October 31, said that the Newcastle Infirmary had "a better prospect than for many years." In other words, the excess of expenditure over income for the ten months of this year is £2,259, against £4,354 for the same period in 1909. The daily average number of patients and officials was 670, "the high-water mark," Mr. Sutton said, of admissions, which caused heavy expenditure, there being so many to keep. In answer to Mr. Jeffery, it was stated that a committee was now sitting to consider the interests, the wages, and the hours of the menial employés of the institution. We understand that both wages and hours have been recently under revision.

**Newcastle Infirmary.**

The festival dinner of King's College Hospital this year was a social and a financial success, for £1,672 was announced in subscriptions that evening. Mr. Walter Long presided, and the Hon. W. F. D. Smith, chairman of the hospital, took occasion to remark, as The Hospital has done often, on the value of an efficient almoners' department, and how its establishment at King's assured the public against the abuse of the out-patient department, which in many hospitals all over England had been the subject of much criticism just lately. In South London, Mr. Smith went on, the most complicated part of their new building had been put up, and the new year would see a fresh campaign to be crowned with a dinner, at which Lord Selborne had consented to take the chair. "I do not think it would be wise," Mr. Smith added, "to appeal for less than £150,000, which I hope will soon be subscribed." The speech was listened to by a distinguished company, which included: The Hon. W. F. D. Smith, chairman of the hospital; Admiral of the Fleet Sir Gerard Noel; Major-General Sir Hugh McCalmont; Sir Walter Howell; Sir William Allchin; Mr. A. W. Rowden, K.C.; and the Rev. Dr. Headlam, Principal of King's College.

**King's College Hospital.**

It was a very pleasant task which lay before the special meeting which the governors of the Royal South Hants and Southampton Hospital summoned the other day, namely, the receiving of the report on the plans for the new building, and of the news that Miss Burrell, of Botley, had come forward with the splendid gift of £7,000 from herself. In other words, Miss Burrell has undertaken practically the entire cost of the new out-patients' department, which is, let us add, her memorial to the late Mr. Burrell, her brother. Colonel Willan, J.P., then expressed the thoughts of those present by recalling the long period, some thirty years, during which Mr. and Miss Burrell had befriended the hospital. He had always said the policy of the Committee should be to go ahead and do all they could to keep the hospital up to date, and trust to Providence. Providence had already been extraordinarily kind to them. The governors then appointed Mr. Courtenay Wilson, Colonel Perkins, and Mr. Bayford as trustees, and Mr. Wilson and Colonel Perkins were also appointed trustees of investment. The plans then were passed, and authority was given for the work on the new building to go forward.

**Royal South Hants Hospital.**

The Birmingham General Hospital has issued an appeal for money to pay off its debt of £11,427. We take the leading points from the President's (Lord Newport) letter: "The main reasons which make it necessary in this case to appeal to the generosity of the public are these: Firstly, the district over which the hospital attracts patients is a very wide one indeed, and yet the support which it receives comes from only a small portion of that district; and secondly, the increase of work, more particularly of operations, has been very large of recent years, necessitating a heavy increase in expenditure. In ten years the operations (many of them of a most serious and important character) have risen from 1,702 to 3,929 in number. Notwithstanding the great increase in the work and consequent increase in expenditure, the subscription list is gradually diminishing, while owing to easier means of transit patients are coming from wider and wider areas." It must not be forgotten that the General Hospital at Birmingham is the oldest, and the largest hospital in the

**Birmingham General Hospital.**

Midlands. Birmingham, as their metropolis, will not forget even in the stress of an election an institution on the proper support of which the health of many voters and non-voters largely depends. As the city with the reputation of accomplishing elections in the shortest time, it should be the first to turn, once they are over, to the support of its own charity.

**Birmingham Hospital for Skin Diseases.**

Nine years ago an electric plant for the treatment of lupus was installed at the Birmingham and Midland Hospital for Skin and Urinary Diseases. In 1907, in spite of such increased accommodation as the hospital was able to give by enlarging the board-room and transforming it, it was felt that the growing importance of the department and the increased numbers attending it made new and adequate housing a necessity. The new department has just been opened by Mr. William A. Cadbury. It has cost £2,000, and now contains three sets of *x*-ray apparatus, but since its earliest days no member of the medical or nursing staffs has suffered from *x*-ray dermatitis. The department contains not merely the rooms for the apparatus, but also one for chemical and bacteriological examinations. Dr. Russell Green has been placed in charge. Mr. W. Johnson presided, and the key of the new department was handed to Mr. Cadbury by the architect, Mr. Thomas Guest. The department is ready, but money is needed for the purchase of radium.

**Wells Cottage Hospital.**

The new Wells Cottage Hospital, Holkham, has been opened by the Earl of Leicester, who, indeed, was asked by Colonel J. E. Groom, J.P., to accept it as a memorial of the late Earl. The hospital was wanted badly, and will supply treatment for a district which is over thirty miles from the county institutions at Norwich and Lynn. The hospital possesses six beds and two cots, and was built at the unanimous wish of the estate tenants. The Hon. Secretary, Mr. Edward Nelson, has been exceedingly busy, and a tribute to his work and activity was given at the meeting, which was attended by a large number. The hospital will be finished and ready by New Year's Day.

